# Recent Advances in Molecular Research in Rice: Agronomically Important Traits

**DOI:** 10.3390/ijms21175945

**Published:** 2020-08-19

**Authors:** Kiyosumi Hori, Matthew Shenton

**Affiliations:** National Agriculture and Food Research Organization, Institute of Crop Science, Tsukuba, Ibaraki 305-8518, Japan

Rice (*Oryza sativa* L.) is the most important food crop in the world, being a staple food for more than half of the world’s population. Recent improvements in living standards have increased the worldwide demand for high-yielding and high-quality rice cultivars [1]. To achieve improved agricultural performance in rice, while overcoming the challenges presented by climate change, it is essential to understand the molecular basis of agronomically important traits related to increasing grain yield, grain quality, disease resistance, and stress tolerance. Recently developed techniques in molecular biology can reveal the complex fundamental mechanisms involved in the control of these agronomic traits [2,3,4]. As rice was the first crop genome to be sequenced in 2004 [5], molecular research tools are well established in rice, and wide-ranging molecular studies using natural variation, mutants, transgenic lines, genome editing lines, and whole-genome analyses, such as genomics, transcriptomics, proteomics, epigenetics, and metabolomics, are increasing our knowledge about factors affecting agronomic traits. These significant advances will enable the development of novel rice cultivars, combining superior agronomic performance with resilience to climate stresses.

As for many other crops, release of the reference genome sequence has facilitated the identification and cloning of genetic factors, such as genes and quantitative trait loci (QTLs) involved in the control of various agronomic traits in rice [5]. The rice genome contains more than 37,000 annotated genes. However, notwithstanding the many research achievements to date, the individual molecular functions of most genes are still unknown. Continuing research efforts in gene functional analysis are necessary to elucidate the genetic networks and molecular mechanisms controlling agronomically important traits. In the Special Issue “Molecular Research in Rice: Agronomically Important Traits”, we collected several recent studies that identified genetic factors and revealed their molecular contributions to rice agronomic traits under various cultivation conditions.

Increasing grain yield is a major objective in breeding programs, because of the need to meet the increased demand for rice fueled by population growth. Changes in plant architecture, such as in grain shape, grain number per panicle, plant height, number of tillers, leaf size, and leaf angle, have been associated with improved grain yield. Seo et al. showed that the *OsbHLH079* gene is associated with the control of leaf angle and grain shape via brassinosteroid biosynthesis and signaling pathways [6]. Gull et al. focused on grain shape and grain weight, and they estimated the genetic effects of each allelic combination among seven genes—*GS2*, *GS3*, *GS5*, *GW5*, *GS7*, *SLG7*, and *GW8*—in improving grain length, grain width, grain thickness, and thousand grain weight [7]. Jiang et al. identified a semi-dwarfism gene, *OsCYP96B4*, which affected the content of γ-aminobutyrate (GABA), amino acids, saccharides, and other secondary metabolites in rice plants [8]. Yu et al. detected eight QTLs underlying heterosis in traits, including days to heading, grain yield, and plant height between the two rice subspecies *O. sativa* ssp. *indica* and *O. sativa* ssp. *japonica* [9]. Zhang et al. found a large set of genes that are differentially expressed at the shoot apical meristem in response to nitrogen application, which regulated the number of tillers and panicles in rice plants [10]. Gao et al. identified that the *OsEWL4* gene was a regulator of tiller number and plant biomass (yield of above-ground parts in rice plants) by using targeted mutations of eight rice *FLW* genes using the CRISPR/Cas9 system [11]. Fan et al. produced overexpression and knockdown transformants of the *OsNAR2.1* gene and revealed that changing expression levels of the *OsNAR2.1* gene altered global methylation at the whole genome level and the phenotypes of plant height and grain yield at the plant level [12]. These novel technologies are powerful tools to understand the molecular basis of increasing rice yields.

It is also important for the understanding of the genetic basis of yield components to characterize genes that function in chloroplast development and chlorophyll degradation during leaf greening and senescence. Shim et al. reported that the grain shape gene *GW2* was responsible for the leaf senescence QTL *qGC2*. They revealed that *GW2*, encoding a RING-type E3 ubiquitin ligase, controlled both grain shape and chlorophyll content by the transcriptional regulation of the cytokinin, brassinosteroid, auxin, and abscisic acid (ABA) phytohormone signaling pathways [13]. Zhang et al. showed that the *OsCAF1* gene encodes a chloroplast RNA splicing and ribosome maturation domain (CRM) protein and plays a key role in chloroplast development in rice [14]. Zhao et al. investigated the effects of low temperature treatment on the greening of rice seedlings. Decreased chlorophyll content was caused by the inhibition of δ-aminolevulinic acid and the suppression of conversion from protochlorophyllide into chlorophyll [15].

Rice consumers pay particular attention to high grain quality, and grain quality largely determines the market price of rice grains. Therefore, grain quality is an important trait alongside grain yield in rice. Wu et al. detected a total of 14 QTLs for protein content in rice grains. A stably inherited QTL, *qGPC1-1*, was delimited within an 862 kbp genome region on the high-density linkage map [16]. These research efforts are helpful in dissecting the genetic basis of grain components and improving rice grain quality in future breeding programs.

Besides grain yield and quality, traits in resistance to biotic and abiotic stresses are of paramount importance as climate change tests the resilience of rice production systems. Recent studies have provided important knowledge of biotic interactions between rice and pathogens. Resistance genes against blast disease caused by *Magnaporthe oryzae* and bacterial blight disease caused by *Xanthomonas oryzae* pv. *oryzae* have been genetically identified. Furthermore, there are many studies investigating the molecular mechanisms of rice immune responses, such as receptor-mediated pathogen recognition, host immune signaling, and pathogen effector-mediated susceptibility. In this Special Issue, Kanda et al. focuses on the *BSR1* gene, which encodes a receptor-like cytoplasmic kinase subfamily VII protein and concludes that the hyperactivation of microbe-associated molecular pattern (MAMP)-triggered immune responses conferred robust *BSR1*-mediated broad spectrum resistance to fungal and bacterial pathogens [17]. Liu et al. reports that the *OsAAA-ATPase1* gene enhanced the expression of pathogenesis-related genes, salicylic acid (SA)-mediated defense responses, and resistance to rice blast fungus [18]. Liang et al. found a novel allele of the durable blast resistance gene *pi21*, which was associated with basal resistance to rice blast disease. They detected four QTLs for basal resistance, one of which was identified as a novel haplotype of *pi21* [19]. Hsu et al. developed a rice line, pyramiding five bacterial blight resistance genes: *Xa4*, *xa5*, *Xa7*, *xa13*, and *Xa21*. The pyramiding line showed not only high levels of resistance to bacterial blight disease, but also increased grain yield and grain quality [20].

Abiotic stresses, including high temperature, low temperature, and saline conditions, are an important issue for rice cultivation because they cause significant decreases in yield and grain quality. Various genes have been identified, cloned, and functionally characterized with the aim of overcoming these stresses and protecting rice plants. Jiang et al. detected 11 QTLs for low temperature germination ability and identified the *DEP1*, *qLTG3-1*, and *OsSAP16* genes as being responsible for three QTLs, proposing a novel candidate gene for another QTL, *qLTG6* [21]. Yuan et al. detected a total of 36 QTLs for seed dormancy and germination behavior and identified a candidate gene for one major effect QTL, *qDOM3.1*, which was involved in an ABA signaling pathway [22]. Sheteiwy et al. evaluated the priming effects of GABA on seed gemination and seedlings under saline and osmotic stress conditions. The salinity and osmotic stress treatments resulted in obvious increases in the expression of *OsCIPK* genes that were associated with changes in cell ultra-morphology and cell cycle progression [23]. Liu et al. identified differentially expressed genes in anthers in a thermotolerant rice cultivar. The expression and polymorphism analysis of the *OsACT* gene suggested that it may be involved in high temperature tolerance in rice cultivars [24]. Finally, Schaarschmidt et al. investigated alterations in more than 140 metabolites and in agronomic traits, including grain yield and plant height under high night temperature stress conditions [25].

The significant studies mentioned here demonstrate the importance of the research community in understanding and explaining the molecular genetic basis of agronomically important traits in rice. To develop novel rice cultivars, showing climate resilience and strong agronomic performance in the future, we have to identify further important genes, elucidate their molecular functions, and design desirable genotypes based on the individual and interaction effects of important genes. In addition to the traits described in this Special Issue, there are many other agronomically important traits that should be explored using molecular genetic and biological research. The sharing of experimental results among researchers facilitates the development of new rice cultivars in future rice breeding programs. Thus, these research efforts are necessary to address the increasing food supply and security problems around the world.
